# ABCs of base therapy in neonatology: role of acetate, bicarbonate, citrate and lactate

**DOI:** 10.1038/s41372-024-02169-x

**Published:** 2024-11-12

**Authors:** Gagandeep Dhugga, Deepika Sankaran, Satyan Lakshminrusimha

**Affiliations:** 1 Department of Pediatrics, University of California, Davis, CA USA; 2 Division of Neonatology, Department of Pediatrics, University of California, Davis, CA USA

**Keywords:** Paediatrics, Metabolism

## Abstract

Metabolic acidosis is common in preterm and term newborn infants and may be attributed to a variety of etiologies, potentially requiring base therapy such as acetate or bicarbonate. However, concerns exist regarding potential harm of sodium bicarbonate due to intracellular acidosis, fluctuations in cerebral blood flow, and osmolar load with rapid infusions, with no improvement in survival when used during resuscitation. Alternative approaches to correct metabolic acidosis include the addition of acetate in parenteral nutrition, intravenous lactated Ringer’s (LR) solution, and use of oral citrate. Current guidelines focus on addressing the underlying cause of acidosis, reserving the use of sodium bicarbonate (NaHCO_3_) for severe cases requiring acute correction, LR instead of saline for volume boluses and using acetate or citrate for slow correction to stabilize acid-base status. Further research is necessary to better understand the efficacy and safety of acetate, NaHCO_3_, and other base sources in treating metabolic acidosis in neonates.

## Introduction

Metabolic acidosis is common in preterm and term newborn infants secondary to various conditions such as asphyxia, sepsis and persistent pulmonary hypertension of the newborn (PPHN). Pediatricians have attempted to address metabolic acidosis by adding base to intravenous solutions for almost a century [1]. Effective management of metabolic acidosis and optimization of intravenous solutions for use in pediatrics has an interesting history. In this article, we have reviewed the history of use of base in newborns, the potential benefits and harms of adding base to correct metabolic acidosis in newborns and the current knowledge gaps.

### History of intravenous solutions and base in Pediatrics

Sydney Ringer (1834–1910) worked for a short period of time at the Hospital for Sick Children in Great Ormond Street and Brompton Hospital for Diseases of the Chest. When his senior colleague, Sir William Jenner reprimanded him for holding posts in two hospitals, Ringer (unfortunately) resigned from the Children’s Hospital. Ringer was interested in the action of various salts on cardiac function. While experimenting on a frog’s heart [2–4], his assistant mistakenly substituted tap water (supplied by the New River Water Co which had 278.6 ppm of solids including 38.9 ppm of calcium) for distilled water [5, 6]. Being an astute observer, Sydney Ringer realized that the presence of small quantities of inorganic salts of sodium, potassium, calcium and chloride had an impact on cardiac function [6]. Ringer’s solution was formulated as a result of this discovery. He further emphasized the importance of small quantities of salt by showing that fish could not survive in distilled water but adding minute quantities of river water was sufficient to keep them alive.

**Fig. 1:**
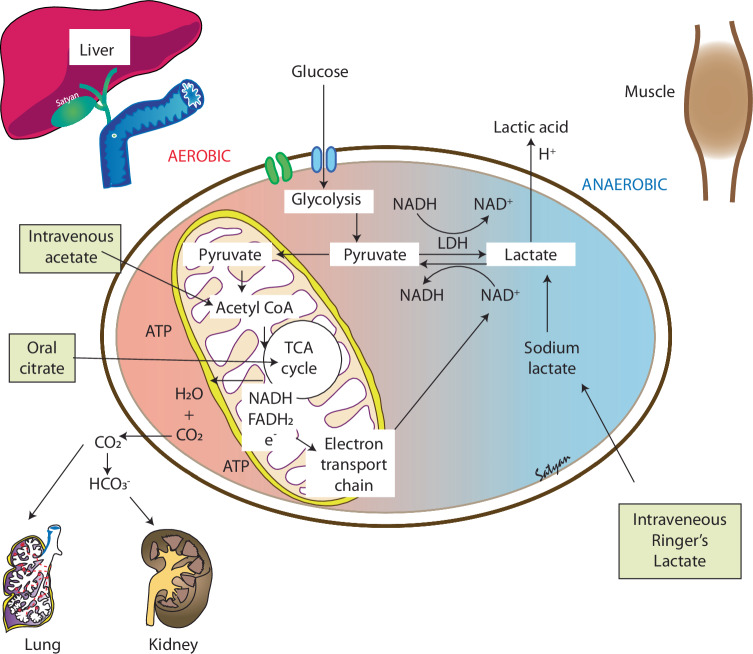
Mechanism of action of bases in treatment of metabolic acidosis. Administration of Ringer’s lactate solution is followed by the conversion of lactate to pyruvate by lactate dehydrogenase. Pyruvate is then converted to acetyl CoA, which enters the tricarboxylic acid (TCA) cycle (also known as the Krebs cycle), ultimately resulting in the generation of HCO_3_^−^ that enters the circulation and CO_2_ that is exhaled by the lungs. The administration of intravenous acetate and oral citrate provides substrates for acetyl CoA and citric acid in the TCA cycle, respectively. Copyright Satyan Lakshminrusimha.

Alexis Frank Hartmann (1898–1964) was an American pediatrician and clinical biochemist. He identified the need for alkali therapy to correct acidosis in children and advocated the use of sodium lactate as an option to reduce chloride concentrations. Sodium lactate would normally convert to NaHCO_3_ over a 2-h period (Fig. 1) and a liter of 1/6 M sodium lactate had the same acid neutralizing effect of 290 ml of 5% NaHCO_3_. By addition of lactate to the original Ringer’s solution, the Hartmann’s solution or “lactated” Ringer’s solution (LR) had the following composition: 131 mEq/L of sodium, 5 mEq/L of potassium, 4 mEq/L of calcium, 29 mEq/L of lactate and 111 mEq/L of chloride [1]. It is important to remember that the LR solution or the Hartmann solution as we currently use it, was formulated by a pediatrician with an intention to correct metabolic acidosis in sick children [7]. Sodium bicarbonate is hard to sterilize, interacts with calcium and produces rapid fluctuations in pH and is not an ideal constituent of bolus or maintenance solutions. However, sodium lactate when added to the hypotonic Ringer’s original solution was stable and allowed slow conversion to bicarbonate in the liver (Fig. 1) [8].

Although, NaHCO_3_ was initially introduced as a therapeutic agent for the management of malignant cholera [9], the widespread use of intravenous NaHCO_3_ in the neonatal intensive care unit (NICU) began during the 1950s and 1960s. Sodium bicarbonate was first commercially produced in the 1950s, and its use in premature neonates became prevalent for preventing azotemia, elevations in serum potassium levels, as well as for correcting metabolic acidosis (Usher-regimen) [10]. However, Corbet et al observed a slight increase in deaths in a controlled trial of NaHCO_3_ in high-risk preterm infants [11]. Usher himself recognized the association between rapid NaHCO_3_ therapy and intraventricular hemorrhage (IVH) in preterm babies [12]. Wigglesworth also suspected a direct link between alkali use and IVH in infants with hyaline membrane disease [13].

**Fig. 2:**
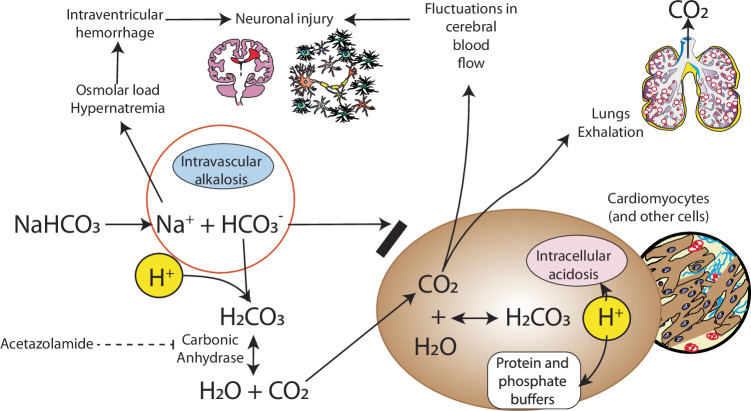
Metabolic effects or rapid administration of intravenous sodium bicarbonate. Although intravascular correction of pH and alkalosis occurs, due to better diffusion of carbon dioxide into the cells (compared to bicarbonate ion), intracellular acidosis can occur, especially in cardiomyocytes. High osmolar load and fluctuations in cerebral blood flow can contribute to intraventricular hemorrhage. Copyright Satyan Lakshminrusimha.

Animal studies showed that NaHCO_3_ and glucose infusions prolonged survival after asphyxia and possibly lessened the degree of cerebral injury [14]. These studies, along with others, suggested the utility of NaHCO_3_ in neonatal resuscitation and was listed in the Medications chapter of the initial editions of the Textbook of Neonatal Resuscitation. Starting in the 1970s, other studies emerged showing that NaHCO_3_ may not be beneficial and in fact may be harmful. For example, a study by Ostrea et al. showed the paradoxical effect of increasing PCO_2_ and decreasing intracellular pH after administration of NaHCO_3_ in the absence of adequate ventilation (Fig. 2) [15]. In 2006, a Cochrane review of randomized controlled trials (RCT) found insufficient evidence to determine whether the infusion of NaHCO_3_ during neonatal resuscitation reduces mortality and morbidity [16]. Currently, NaHCO_3_ is not recommended during neonatal resuscitation but is used selectively and sparingly in the NICU [17–19].

The use of acetate as a buffer to correct metabolic acidosis crept into neonatal practice towards the end of the 20^th^ century. Use of sodium acetate is associated with higher pH and reduced need for NaHCO_3_ in preterm infants [20, 21]. Similar to sodium lactate, acetate has to be metabolized to carbon dioxide through the Krebs cycle and contributes to HCO_3_ formation (Fig. 1).

### Acid-base homeostasis mechanism

**Fig. 3:**
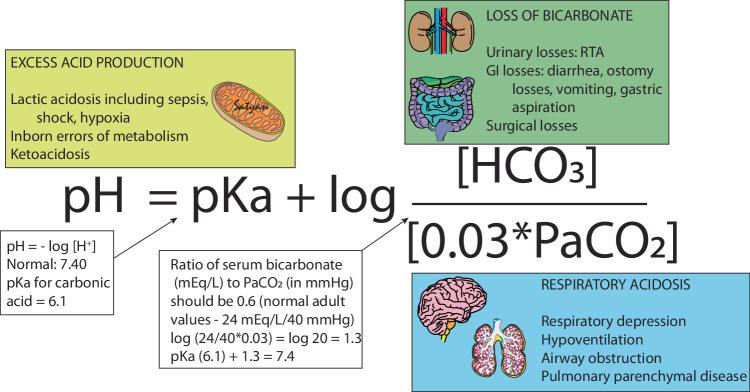
Henderson–Hasselbach equation. The Henderson–Hasselbalch equation helps assess the acid-base status of a patient and determine whether respiratory or metabolic factors are contributing to an acid-base imbalance. The yellow (excess acid production), green (loss of bicarbonate), and blue (respiratory acidosis) boxes depict the common causes of acidosis in newborns.

Acid-base homeostasis is tightly regulated by neonates through several mechanisms including buffer systems, respiratory and renal regulation [5]. The etiologies for metabolic acidosis (Fig. 3 and Table 1) include, a) loss of base (gastrointestinal- including ostomy losses or renal, or post-operative losses), b) excessive intake of H^+^ ions beyond what the kidneys can eliminate, c) excessive generation of H^+^ ions from abnormal cellular metabolism, especially lactic acidosis in hypoxia and sepsis. In critically ill infants, severe tissue-level hypoxia secondary to either poor perfusion or oxygen delivery can shift metabolism towards anerobic glycolysis with the generation of H^+^ ions and lower ATP production, and subsequent production of lactic acid [22].

**Table 1:** Neonatal metabolic acidosis mnemonic.

Neonatal metabolic acidosis mnemonic *“ACID SHOCK”*	
A	Asphyxia
C	Cardiac disease (left sided obstruction, cyanotic heart disease, cardiac failure, PDA)
I	Infection (Sepsis), Inborn errors of metabolism
D	Diarrhea
S	Shock
H	Hypoxia
O	Ostomy loss
C	Chloride excess/carbonic anhydrase inhibitors
K	Kidney Losses (distal/proximal RTA)

The various factors that can cause a neonate to have metabolic acidosis could easily be remembered with the mnemonic ACID SHOCK.

A serum pH in the range of 7.35–7.45 provides the ideal environment for cellular metabolism, making the maintenance of a stable pH critical for homeostasis. Buffer solutions resist changes in pH when acid or alkali are added due to their ability to absorb or release H^+^ ions. The three main buffer systems are the HCO_3_, phosphate and protein systems [23]. The protein and phosphate systems are intracellular, where extracellular H^+^ is exchanged for sodium and potassium ions. The bicarbonate system is extracellular and consists of a mixture of carbonic acid (H_2_CO_3_) and NaHCO_3_. This extracellular buffer system works by combining excess H^+^ with HCO_3_^−^, forming H_2_CO_3_, which dissociates to give CO_2_ and H_2_O (Fig. 2). The CO_2_ is then excreted from the lungs through exhalation (${H}^{+}+{HC}{O}_{3}^{-}\,\longleftrightarrow {H}_{2}{{CO}}_{3}\longleftrightarrow {H}_{2}O+C{O}_{2}$). As shown in the Henderson–Hasselbach equation (Fig. 3), exhalation of CO_2_ shifts the reaction to the right allowing HCO_3_ to play an important role in buffering the H^+^ ion and reducing acidosis.

### Interpretation of base excess on an arterial blood gas

Base excess on an arterial blood gas can help clinicians understand the overall acid-base balance in a neonate. However, base excess should not be interpreted in isolation when determining acid-base status of an infant. Base deficit is a theoretical estimate that is calculated by a blood gas analyzer utilizing measured values of pH, PaCO_2_ and hemoglobin. Some blood gas machines report two different values for base deficit or base excess: blood and ECF. Base deficit in blood is the amount of base that needs to be added or extracted (i.e., base excess) to a liter of blood to shift the plasma pH to 7.4 at a temperature of 37 °C and PaCO_2_ of 40 mm Hg [24]. As blood gas machines calculate base excess using measured values of pH and PCO_2_, acute changes in PCO_2_ without changing serum bicarbonate can alter calculated base excess values. For example, a term infant with perinatal acidosis may have an ABG with a pH of 7.1, PaCO_2_ of 40 mmHg, bicarbonate of 12 mEq/L and a calculated base excess of −15.7 mEq/L. If this infant develops acute endotracheal tube obstruction with an increase in PaCO_2_ to 60 mmHg without any change in serum bicarbonate, the pH decreases to 6.92 and calculated base excess will be −18.2 mEq/L.

On the contrary, the standard base deficit in the extracellular fluid (ECF) consists of blood volume diluted with the interstitial fluid with a standardized hemoglobin concentration of 5 g/dL (a low concentration of hemoglobin is utilized in this calculation to represent dilution by ECF). This base deficit in ECF refers to the metabolic (or non-respiratory) component of base deficit, as it is minimally affected by changes in PaCO_2_ due to dilution by interstitial fluid, compared to the base deficit in blood that is increased by an increase in PaCO_2_ and the conversion of CO_2_ to HCO_3_ by erythrocytes [24].

Various factors can affect the reported value of base excess, including inconsistencies between different equations used to calculate base excess, such as the Siggaard-Andersen equation (=0.9287 × (HCO_3_ − 24.4 + 14.83 (pH − 7.4)) and the Van Slyke equation (=cHCO_3_ − 24.4 + (2.3 × hematocrit + 7.7) × (pH − 7.4) × (1 − 0.023 × hematocrit)), as well as clinicians’ not knowing which equation is used by their clinical labratory [25]. Additionally, the unique aspects of fetal acid-base physiology, which are not accounted for in these equations, can lead to inaccurate values [25]. For example, these equations do not account for acid-base status of newborns at birth and rely on adult acid-base physiology norms (pH 7.4, pCO_2_ 40 mmHg and HCO_3_ levels ranging from 24–24.8 mmol/L) as well as adult hemoglobin characteristics [25]. This approach fails to reflect the higher hemoglobin levels found in newborns (~17 g/dL in term newborns compared to ~12 g/dL in adults) and does not consider properties of fetal hemoglobin [25]. Hence it is important to check bicarbonate level on a serum chemistry sample in addition to base deficit on a blood gas before deciding to correct acidosis.

In infants with perinatal asphyxia, metabolic acidosis with pH <7 or base deficit >−12 is included as an essential criteria for the diagnosis of moderate-to severe HIE, along with early onset of encephalopathy, multisystem dysfunction, and exclusion of other etiology such as trauma, coagulation disorders, metabolic disorders and genetic causes [26]. Furthermore, Puthuraya et al demonstrated that time to recovery of base deficit was observed to be longer across the severity of HIE in a cohort study [27]. It is critically important that the umbilical arterial cord blood sample is utilized rather than umbilical venous to assess the base excess, as the CO_2_ is effectively cleared by the placenta in the blood returning to the fetus in the umbilical vein and the metabolic component is overestimated due to the lower PCO_2_ resulting in falsely elevated base excess [24].

### Normal Saline vs. LR for neonatal resuscitation

Previous editions of the textbook of neonatal resuscitation recommended the use of normal saline or LR as a crystalloid for volume bolus. The more recent editions have eliminated LR as a choice. Compared to normal saline, LR is more physiological with a sodium and chloride concentration like that of plasma. The pH of normal saline is significantly lower, contributing to hyperchloremic metabolic acidosis. However, LR is not as commonly available as normal saline in the delivery room, and its use may transiently increase lactate levels (by ~0.27 mM/L following 10 mL/kg of LR). Ringer’s lactate has small quantities of calcium that can precipitate with simultaneous use of blood products. While saline is the preferred solution during delivery room resuscitation, we prefer to use LR to sodium chloride during the post-resuscitation phase, especially in the presence of hyperchloremic metabolic acidosis [28–30].

### Bicarbonate is currently not recommended for neonatal resuscitation

Currently, the use of NaHCO_3_ is not routinely recommended as part of neonatal resuscitation. This is in part due to limited evidence of benefits in improving short-term outcomes following neonatal resucitation [4, 31, 32]. The only placebo-controlled RCT that evaluated use of NaHCO_3_ during neonatal resuscitation found no difference in survival to discharge or neurological outcome at discharge [32]. Moreover, infants who received NaHCO_3_ had a trend towards worse outcomes of encephalopathy, cerebral edema and increased need for inotropes. In particular, there is no benefit in correcting metabolic acidosis secondary to HIE with NaHCO_3_, nor is there evidence in treating respiratory acidosis with NaHCO_3_ when CO_2_ cannot be effectively eliminated from the lungs due to poor ventilation [15, 33, 34]. Given this background, current American Academy of Pediatrics- Neonatal Resuscitation Program (NRP) guidelines do not include routine NaHCO_3_ administration during the acute resuscitation phase, focusing instead on optimal ventilation techniques, chest compressions, and the use of intravenous epinephrine/ volume expanders. In 2000, the guidelines further stated that “the hyperosmolarity and CO_2_ generating properties of NaHCO_3_ may be detrimental to myocardial or cerebral function (Fig. 2)” [35, 36]. However, according to the European resuscitation guidelines, the use of sodium bicarbonate can be considered in prolonged resuscitation with adequate ventilation to reverse intracardiac acidosis, with a dosing recommendation of 1–2 mmol/kg administered by slow infusion [37].

### Bicarbonate use to treat metabolic acidosis in the NICU

Metabolic acidosis can increase mortality in critically ill newborns by causing hemodynamic instability, arterial vasodilation, lowering myocardial contractility, reducing cellular oxygen supply and mitochondrial oxygen consumption as well as impairing catecholamine responsiveness [17, 38, 39]. A golden rule in the NICU for treating metabolic acidosis is to address the underlying cause (e.g. sepsis, hypovolemia, hypoxia, renal and/or gastrointestinal losses) [17, 18, 33, 35]. Reducing acid production by optimizing the delivery of oxygen to tissues is the ideal treatment for acidosis secondary to tissue-level hypoxia. However, in critically ill neonates such as those with critical congenital heart disease, severe PPHN or septic shock, severe metabolic acidosis may be ominous and may warrant correction while the primary etiology is being addressed. Acidosis may contribute to hemodynamic instability by reducing myocardial contractility, predisposing to arrhythmia, inducing vasodilation, and impairing the response to catecholamines by reducing cellular calcium influx, increasing extracellular potassium, and depleting pituitary vasopressin reserves [40].

The presence of severe metabolic acidosis with compensated respiratory alkalosis and hypocapnia in patients with birth asphyxia and HIE deserves further discussion. Infants with perinatal metabolic acidosis have tachypnea due to HIE and Kussmaul respirations with low PaCO_2_ levels [41]. Low PaCO_2_ levels in patients with HIE are associated with increased risk of death or disability at follow-up [42, 43]. Some of these infants are on minimal or no respiratory support. Infants with HIE with absent gag reflex might need an endotracheal tube to protect their airway. In such patients, ventilator adjustments such as low rate, increasing trigger sensitivity (making it difficult to trigger ventilator inflation), decreasing pressure support, increasing termination cut-off for flow cycling to shorten pressure supported breaths and use of sedation (and in extreme situations, use of muscle relaxants) may assist in increasing PaCO_2_. If these measures fail, a slow infusion of a small dose of NaHCO_3_ (1 mEq/kg) may correct metabolic acidosis and increase PaCO_2_. The effect of such small doses of NaHCO_3_ in HIE and hypocapnia on cerebral blood flow, short- and long-term outcomes needs further investigation.

Currently, in the United Kingdom, a study is underway to examine the short-term and long-term health and developmental effects of NaHCO_3_ use in preterm neonates with metabolic acidosis [44]. This study aims to recruit 3764 pre-term infants born between 23^+0^−30^+6^ weeks gestational age in about 45 NICUs in the U.K. over a 3-year period who have metabolic acidosis [44]. The infants will be randomized into two groups: one group will receive NaHCO_3_ for episodes of metabolic acidosis, while the other group will not receive NaHCO_3_ for metabolic acidosis [44]. The primary outcome is survival to discharge without major morbidities including bronchopulmonary dysplasia, retinopathy of prematurity (ROP) requiring treatment, severe IVH or periventricular leukomalacia, late onset sepsis, severe NEC and need for major surgery. These infants will be followed to assess survival without moderate-to-severe neurodevelopmental impairment at 2-years postnatal age corrected for prematurity [44]. Although the study does not set specific dosages of NaHCO_3_ and allows clinicians to use dosing per their unit protocol, it does recommend using the formula: Mmol of NaHCO_3_ = (0.3–0.6) × weight (kg) × base deficit (mmol/L) over 30 min to 4 h for NICUs without set dosing guidlines [44]. This study will be valuable in understanding the effects of NaHCO_3_ in the treatment of metabolic acidosis.

### Current use of sodium bicarbonate in the NICUs

A international survey completed in 2023 showed that, out of a pool of 134 physicians, around 91.2% used NaHCO_3_ to treat metabolic acidosis in their neonatal practice [17]. Although there are known adverse effects associated with NaHCO_3_ administration, such as fluctuations in cerebral blood flow, cardiovascular hemodynamics and increased rates of severe IVH in preterm babies, its use remains a common treatment for metabolic acidosis in NICUs worldwide [17–19]. Another survey completed in Italy showed similar results and also pointed out that dosage and time of infusion varied widely across the centers [19]. With no standard guidelines on indications, dosing or speed of infusion, there is a risk of unsafe treatments, highlighting the need to clarify these dosages [19, 35]. Commonly the following equation has been used to determine the base deficit correction: Full Correction Dose (mmol) = 0.3 X base deficit (mmol/L) X the weight (kg) [17]. and a prevailing practice is to only administer half the dose and reassess the need for the remainder [17]. However, this dose may be excessive due to variations in base deficit as explained previously. We prefer the alternate approach to use 1-2 mEq/kg by slow infusions and reassess acid-base status and hemodynamics then repeat the dose as needed [37, 45, 46]. The use of a lower dose could potentially help reduce the risk of hypokalemia, hypernatremia, hypocalcemia, rebound alkalemia and water-sodium overload.

### Effects of bicarbonate administration on the brain

As mentioned previously, a concerning adverse effect of NaHCO_3_ is fluctuations in cerebral blood flow. Rapid infusion of NaHCO_3_ over less than 5 min has been associated with both decreased and increased cerebral blood flow [47–49]. An RCT was performed in Netherlands to study the effects of rapid vs. slow infusion of NaHCO_3_ on cerebral hemodynamics and oxygenation in 29 preterm infants [48]. In this study, it was shown that a slow infusion of NaHCO_3_ is preferred to minimize fluctuations in cerebral hemodynamics as a rapid infusion of NaHCO_3_ increased cerebral blood volume [48]. A retrospective study by Katheria et al showed that infusion of NaHCO_3_ over 30-minutes administered in 36 preterm infants (23–31 + 6 weeks gestational age) in the first 24 h after birth was associated with increased pH, decreased base deficit and PaCO_2_ and increased cerebral oxygenation without an increase in oxygen extraction or cardiac output [49]. However, these patients also had a decrease in systemic blood pressure, so it is unclear if the increase in the cerebral oxygenation, despite no change in cardiac output, was a compensatory reaction to the decrease blood pressure or a direct effect of NaHCO_3_ [49].

### Effect of bicarbonate administration on the heart

In the past, NaHCO_3_ was used during cardiopulmonary resuscitation in cardiac arrest based on the idea that acidemia impairs myocardial performance and attenuates catecholamine-induced increase in blood pressure, heart rate, and cardiac contractility. Thus, correcting the acidosis with NaHCO_3_ might improve outcomes [35]. However, studies do not support the use of buffers during cardiac arrest and have shown deleterious effects on myocardial performance [35]. An abrupt rise in serum HCO_3_ levels following the administration of NaHCO_3_ may cause paradoxical intracellular acidosis [50]. This can be a particular issue in capillary-rich tissues, such as the heart, which can worsen myocardial contractility [33]. However, given this paradoxical effect is seen with an abrupt rise in HCO_3_ concentrations, a slow infusion of NaHCO_3_ in the post-resuscitation period when ventilation is well established can potentially correct extracellular acidosis without causing paradoxical intracellular acidosis and needs to be investigated.

### Bicarbonate replacement for renal/gastrointestinal losses

The use of NaHCO_3_ to replace gastrointestinal or renal losses can be beneficial [33, 35]. Neonates have lower serum HCO_3_ concentrations due to their immature renal compensatory mechanisms (renal tubular acidosis), which limits their ability to maintain acid-base balance [33, 51]. They also have decreased reabsorption of HCO_3_ at the proximal tubule compared to adults [33, 52]. Another way HCO_3_ is lost in neonates is through the gastrointestinal system where it is actively secreted. The pancreas secretes HCO_3_ into the small intestine to help neutralize gastric acid [33]. When infants have diarrhea, ostomy losses or bilious aspirates much of this HCO_3_ is lost. Under these circumstances, replenishing HCO_3_ can be reasonable [33, 35]. In most cases slow replacement with acetate in the TPN is preferred for gastrointestinal losses and oral citrate for renal losses.

### The use of acetate in parenteral nutrition

Preterm infants often started on parenteral nutrition early to help promote their optimal growth. However, their kidneys are functionally immature, which leads to excessive sodium loss in urine and inadequate urinary acidification [21]. Parenteral nutrition can help replace the losses with sodium chloride. However, this can lead to excessive chloride administration, which may worsen metabolic acidosis in the neonate due to hyperchloremia. An interventional study showed improvement in acid-base status by substituting some of the chloride with acetate in the parenteral nutrition [53]. Another study, which was a prospective blinded RCT, showed similar results in decreasing hyperchloremia and improving the acid-base status of preterm neonates with parenteral administration of acetate [21]. To summarize, the addition of acetate in parenteral nutrition can help improve the acid-base status of neonates. Like lactate, acetate is metabolized through the Krebs cycle to generate CO_2_ and form HCO_3_ to buffer acid (Fig. 1).

### Use of bicarbonate and oral citrate in RTA

Renal Tubular Acidosis (RTA) is a condition in which the kidneys fail to acidify the urine, leading to a buildup of acid in the blood resulting in metabolic acidosis. This can occur due to an impairment in HCO_3_ reabsorption, excretion of H^+^, or both [54]. Mild distal RTA or type 1 RTA can occur in premature neonates and can lead to poor growth and bone demineralization if untreated. There are four types of RTA, classified based on clinical presentation and pathophysiologic mechanisms. Treatment of RTA in neonates includes the use of NaHCO_3_, potassium citrate or Sohl solution (sodium citrate and citric acid monohydrate, or Bicitra) to help correct the metabolic acidosis [54, 55]. The dosing of alkali therapy depends on the type of RTA; a mean dose of 3.5 mEq/kg per day is recommended for type I, whereas type II may require more frequent and higher doses, as high as 14 mEq/kg per day [55]. Thus, the use of oral citrate and HCO_3_ therapy for infants with RTA can be useful to avoid complications such as growth restriction and can help improve bone health.

### The use of lactated Ringer’s solution vs. sodium chloride

Lactated Ringer’s solution contains no HCO_3_ but does contain 28 mmol/L of lactate, which is then converted to HCO_3_ in the liver. LR has lower chloride (109 mEq/L vs. 154 mEq/L in normal saline) concentration and a more neutral pH (6.5). In an adult swine model of hemorrhagic shock, administration of normal saline resulted in hyperchloremic metabolic acidosis and dilutional coagulopathy, whereas use of LR elevated lactate without any exacerbation of acidosis [28]. LR is another alternative to normal saline during resuscitation as a volume expander but relies on optimal liver function to generate HCO_3_. A downside of using LR for its base properties is that it contains calcium and potassium. Calcium is often not compatible with many drugs used in the NICU and can cause precipitation.

## Conclusion

In conclusion, base therapy is commonly used in the NICU. Intravenous NaHCO_3_ continues to hold a limited, selective role in neonatal intensive care. Recently, intravenous acetate and oral citrate have replaced intravenous bicarbonate for slow correction of acidosis [33, 35]. Acute onset of persistent metabolic acidosis can be corrected with lower doses such as 0.5–1 mEq/kg administered slowly over 30–60 min with optimal ventilation while the primary etiology is being addressed. The use of NaHCO_3_ in the NICU needs to be further studied. Currently, a large study is underway in the United Kingdom (BASE trial), serving as the first adequately powered trial to evaluate the clinical and cost-effectiveness of NaHCO_3_ treatment for metabolic acidosis in very preterm neonates, considering both short- and long-term outcomes. This study can shed light on the potential benefits of using an inexpensive drug to improve survival and neurodevelopment in neonates [44].
